# Cohesively enhanced electrical conductivity and thermal stability of silver nanowire networks by nickel ion bridge joining

**DOI:** 10.1038/s41598-018-21777-0

**Published:** 2018-03-27

**Authors:** Shang Wang, Yanhong Tian, Chunjin Hang, Chenxi Wang

**Affiliations:** State Key Laboratory of Advanced Welding and Joining, Harbin Institute of Technology, Harbin, 150001 China

## Abstract

A facile method for producing high-performance nickel enhanced silver nanowire (Ag NW) transparent electrodes on a flexible substrate is reported. The modified electroplating method called enhanced nickel ion bridge joining of Ag NWs, which provides a new route for improving the loose junctions in bare Ag NW networks. The sheet resistance of Ag NW electrode drops from over 2000 Ω sq^−1^ to 9.4 Ω sq^−1^ with excellent thermal uniformity after the electroplating process within 10 s. Nickel enhanced Ag NW transparent films are applied on flexible heaters with good thermal stability (165 °C for 2 h) and mechanical flexibility (3500 cycles under 2.5 mm bending radius) after mechanical bending process. Moreover, the mechanism of nickel growth is also confirmed that the nickel electroplating of the Ag NWs obeyed Faraday’s Laws.

## Introduction

In recent decades, low-dimensional materials, such as nanoparticles, nanowires (NWs) and graphene, have exhibited excellent optical, electrical, thermal, and mechanical properties in various fields^1–5^. Among the various nanomaterials, silver nanowires (Ag NWs) are of great interest to researchers due to their high conductivity (6.3 × 10^7^ S m^−1^)^6^, excellent mechanical properties and facile synthetic routes, which give the nanowires potential to substitute for commercial indium tin oxide (ITO)^7^. In addition to their successful applications in optoelectronic devices (e.g., transparent electrodes) and sensors, Ag NW networks have been used in a variety of areas, such as solar cells, transparent heaters, and high-power devices^8–12^, that require the good reliability of the components under large currents and high temperatures.

However, the electrical properties of Ag NW networks are far from those of the bulk material because of the weak connections between Ag NWs^13^. Ag NW networks also suffer from breakdown when exposed to high applied currents and long-term annealing conditions^14^. Theories have been proposed to address these issues, and various methods have been developed, including heat treatment (featuring either a high processing temperature or long treatment time)^15^, mechanical pressing^16,17^, electrochemical ion exchange^18^, vacuum filtration^19^, metal-oxide nanoparticle fusing^20,21^ and plasmonic welding^22^. In general, these methods require that the Ag NW networks have good uniformity before treatment, but the uniformity is difficult to control. Moreover, to prevent the drawbacks associated with the stability of the Ag NWs, hybrid materials that combine NWs with various nanostructures have been investigated^23^. For example, graphene has been widely used in conjunction with Ag NWs for transparent heaters (THs), as graphene itself is also an excellent flexible TH^24,25^. In another work, THs based on a hybrid structure of a polymer and Ag NWs exhibited a transmittance of 82.3% at 550 nm. By applying a 40 V potential to the films, stable temperature of 99 °C was generated within 50 s^26^. The fabrication of Ag NW-based hybrid structures always influences the transparency of the electrodes. In addition, some of the fabrication processes for accessing hybrid structures are complex, which prevents the widespread applications of these hybrids in industry.

Electroplating is mainly used to protect materials that are sensitive to the ambient environment. By using the traditional electroplating method, Ag NW networks can be selectively coated, avoiding unwanted plating of other areas. This electroplated coating can improve the inter-nanowire connections, which are intrinsically weak. In this study, metal nickel (Ni) was chosen as the electroplating coating material since it had good physical and mechanical properties. Through the electroplating process, high-performance Ni-enhanced Ag NW networks could be fabricated. To ensure the reliability of the high-performance Ni-enhanced Ag NW network, the mechanism by which Ni ions enhance the inter-nanowire connections needs to be identified, and the long-term service behaviour, such as electromigration at high temperatures and connectivity between Ag NWs, needs to be investigated.

## Results and Discussion

### Fabrication and Electroplating of Ag NWs Transparent Films

**Figure 1:**
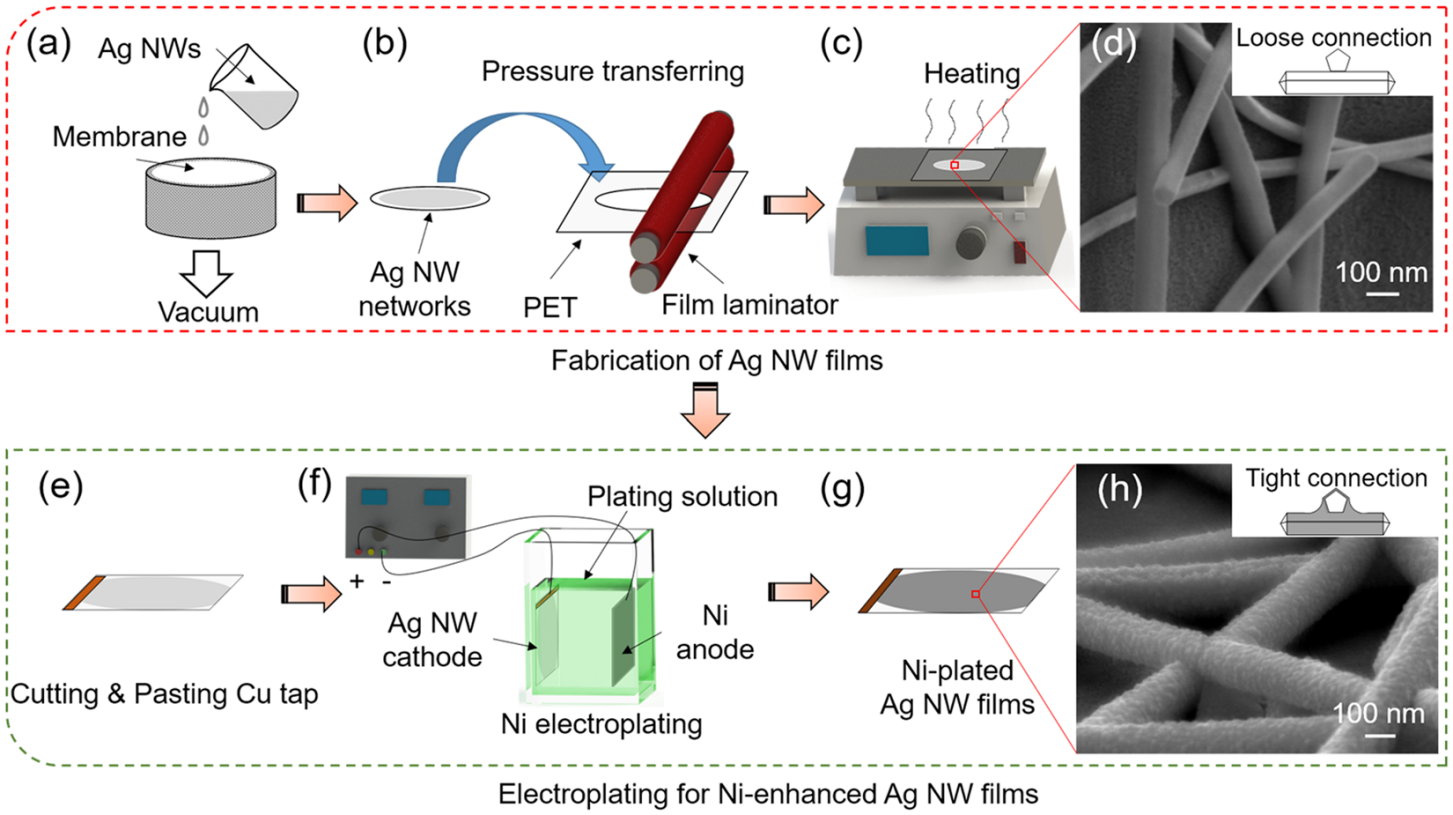
Schematic illustration of transparent heater fabrication. (**a**) Vacuum filtration. (**b**) Pressure transfer process. (**c**) Heating process. (**d**) SEM image of the heated Ag NW network and its connection conditions. (**e**~**g**) Ni electroplating process. (**h**) SEM image of the electroplated Ag NW network and its connection conditions.

Figure 1 shows the entire fabrication process of Ni enhanced Ag NW transparent films. Ag NWs with high aspect ratios (80 μm in length and 80 nm in diameter) were synthesized through a modified polyol method and dispersed in deionized (DI) water. Then, the Ag NW solution was passed through a nitrocellulose membrane by a vacuum filtration system, as shown in Fig. 1a. After filtration, Ag NW networks were coated on the membrane and then were transferred onto a polyethylene terephthalate (PET) film using a laminator (Fig. 1b). The transparency of the untreated films was 90%, and the sheet resistance was over 2000 Ω sq^−1^. After heated at 50 °C for 30 min using a hotplate (Fig. 1c), the sheet resistance of Ag NW films dropped to less than 200 Ω sq^−1^.

However, the heated Ag NW networks were unable to continuously conduct a high current at high temperatures for long periods, especially when used in flexible heaters and high-power devices. The Ag NWs have polygonal cross sections with sharp edges, and as seen in Fig. 1d, they merely stack together, leading to the poor conductivity of the film. To further improve the electrical properties of the Ag NW films and protect the nanowires from electromigration, a thin Ni shell was electroplated on the Ag NW networks. A flowchart of the electroplating process is shown in Fig. 1e–g. The PET film with adhered Ag NWs, referred to as the Ag NW film, was cut into 4 cm × 3 cm coupons. Cu tapes were pasted on the as-prepared Ag NW film for application as the cathode for the electroplating process. The electroplating system consisted of a Ni anode, the Ag NW-based cathode, an electroplating solution and a direct-current (DC) power supply, as shown in Fig. 1f. The electroplating parameters were 100 mA (0.2 A dm^−2^) and 10 s. After the Ni electroplating process, the sheet resistance of the Ni-coated Ag NW films decreased to 9.4 Ω sq^−1^, the transparency dropped to 80%, and the colour of the film changed from light grey to dark grey.

The microstructure of Ni electroplated Ag NWs film was exhibited in Fig. 1h that Ni filled the gaps between Ag NWs forming a tight connection between the individual Ag NWs. Thus, the conductivity of the Ag NW films increased. Furthermore, the junctions between Ag NWs changed from a loose connection to a tight connection because of the Ni electroplating process. Moreover, with the deposition of Ni atoms, the NWs became thick, and their surface became rough. The transmission electron microscopy (TEM) image of the film showed that a rough shell was present on the smooth surface of each Ag NW (Fig. S1a), and the energy dispersive X-ray spectroscopy (EDS) analysis shown in Fig. S1b also confirmed that only Ni and Ag were present. It was concluded that the Ag NW network was connected and coated with thin Ni layer, which was expected to improve the conductivity and anti-electromigration property.

### Performance of the Transparent Heaters

**Figure 2:**
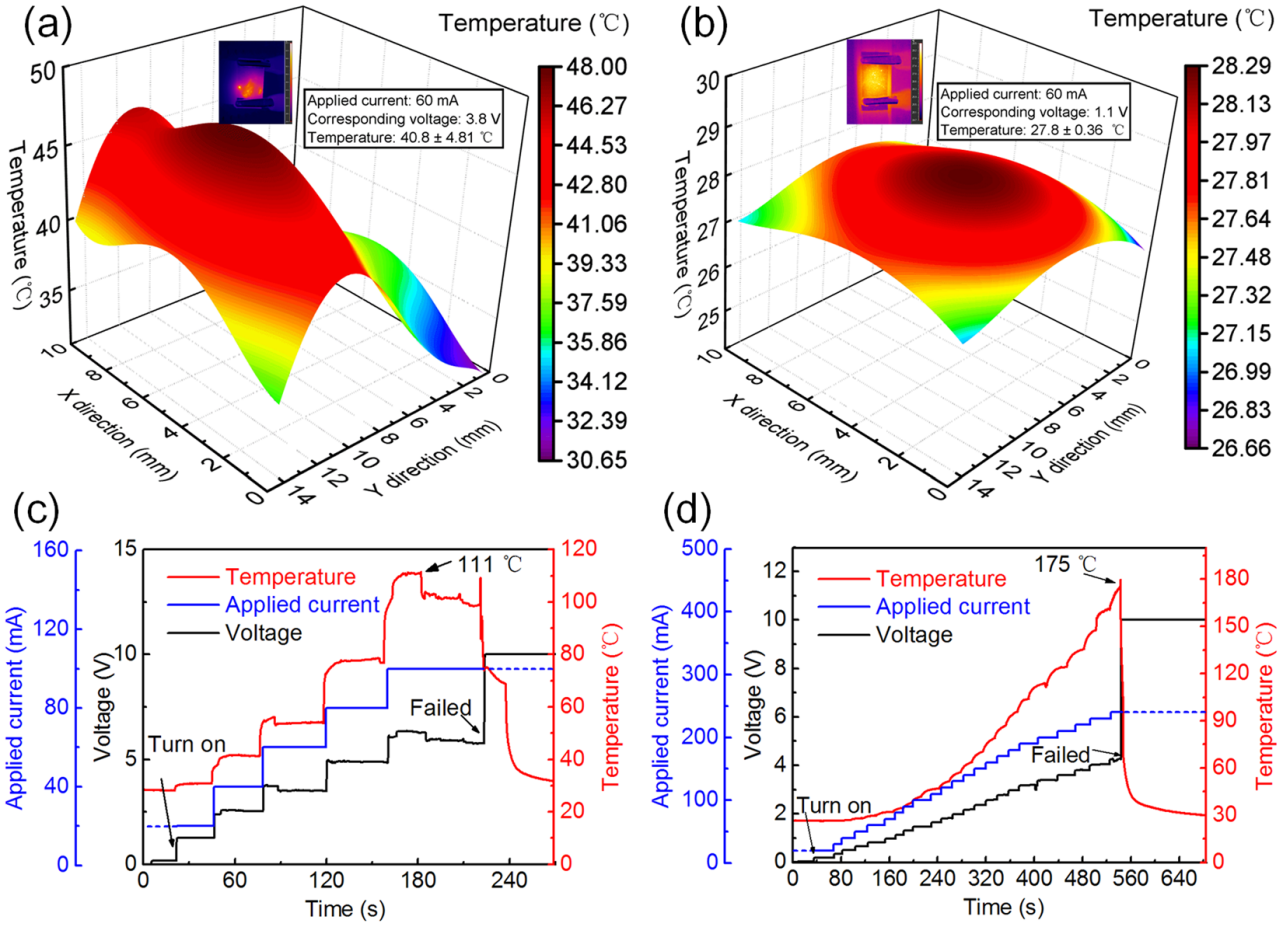
Thermal distributions of (**a**) bare Ag NW and (**b**) Ni-enhanced Ag NW electrodes. Ultimate performance of (**c**) bare Ag NWs and (**d**) Ni-enhanced Ag NWs, respectively. The inset images in (**a**,**b**) are infrared images of the electrodes under a constant applyied current.

To compare the uniformity of the bare Ag NW (bare) and Ni-enhanced Ag NW (plated) networks, the thermal and electrical behaviours of the two types of electrodes with the same size of 2 cm × 1.5 cm were tested. The thermal distributions of the bare and plated electrodes at a current of 50 mA were depicted in Fig. 2a,b, respectively. The average temperature of the bare electrode was 41 °C with a non-uniform thermal distribution. In contrast, the average temperature of the plated electrode reached only 29 °C, and the thermal distribution was relatively uniform. This result indicated that the sheet resistance of the bare electrode was higher than that of the plated electrode and that the network of the bare electrode was not well connected. Ni electroplating enhanced the connection between Ag NWs and made the network more uniform. The thermal and electrical behaviours of the electrodes fabricated with the bare and Ni-plated Ag NWs also differed under incrementally applied currents. The current was increased by 20 mA and 10 mA every 30 s for the bare and plated electrodes, respectively until the electrodes failed, as shown in Fig. 2c,d. The voltage of the Ni-plated electrodes increased linearly with increasing current, and the equivalent electrical resistance was maintained at approximately 18.2 Ω. However, the bare Ag NW electrode exhibited an unstable sheet resistance that varied from 58.1 Ω to 67.3 Ω. Moreover, the Ni-plated electrode had a saturated temperature of 175 °C at an applied current of 240 mA with a maximum voltage of 4.3 V, as opposed to the bare Ag NW electrodes, which only reached a temperature of 111 °C at an applied current of 100 mA with a maximum voltage of 6.3 V.

**Figure 3:**
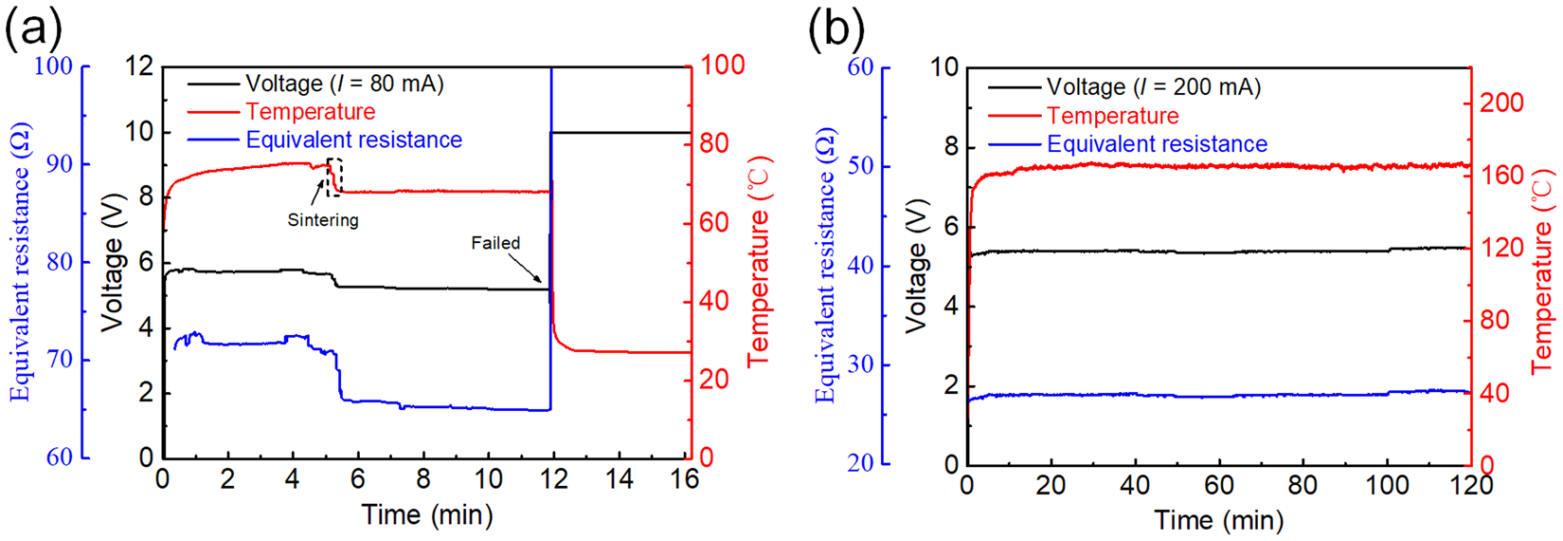
Long-term working stabilities of (**a**) the bare Ag NWs and (**b**) the Ni-enhanced Ag NWs.

To examine the thermal and electrical stabilities of the bare and Ni-enhanced Ag NW electrodes, a constant current was applied to the samples for 2 h, as shown in Fig. 3a,b. The bare Ag NW electrode exhibited good stability at a temperature of 75 °C with a subtle fluctuation prior to 5 min. However, the temperature suddenly dropped to 68 °C at 5 min, and this temperature was then maintained up to 12 min, at which point the sample finally broke down due to the instability of the nanostructures at relatively high temperatures for the long-term annealing^14^. The temperature drops from 75 °C to 68 °C was attributed to the annealing of the Ag NWs in the environment of joule heating^27^ because annealing can lead to decrease in the electrical resistance and the total power. In contrast to the bare Ag NW electrode, the plated electrode maintained a temperature of 165 °C with a stable electrical resistance during the test. Hence, the Ni coating undoubtedly enhanced the stability of the Ag NW networks far above that of the bare Ag NW network in the harsh test environment.

**Figure 4:**
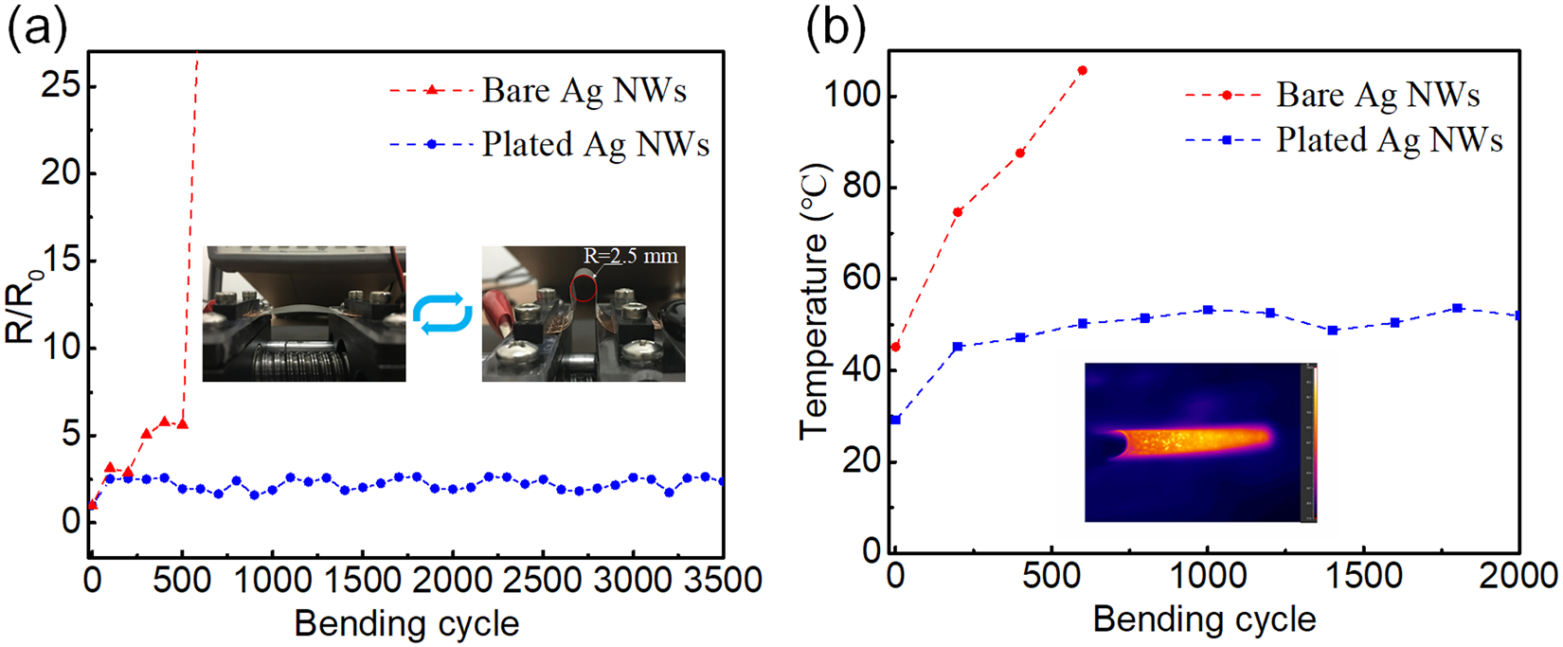
(**a**) Bending tests for determining the mechanical stability of the bare Ag NWs and the Ni-enhanced Ag NWs. (**b**) Variations in the temperature of the heaters during the bending cycles.

The electrical and temperature stability under bending status is also important for the application of heaters on wearable electronics. The mechanical stability of the Ni enhanced Ag NWs heater, and bare Ag NW heater was examined with bending radius of 2.5 mm as shown in Fig. 4a. It was demonstrated that no apparent increase in R/R_0_ was observed even over approximately 3500 cycles for the Ni-enhanced Ag NW heater since the Ag NWs were firmly connected by the electroplated Ni. In contrast, a breakdown occurred after 600 cycles for the bare Ag NWs, as indicated by a dramatic increase in R/R_0_. Moreover, the Ni-enhanced Ag NW-based heater also exhibited excellent working stability after cycled bending process (Fig. 4b). For an applied input current of 60 mA, the temperature should increase due to the increasing resistance of the heater according to Joule’s law. The output temperature of the plated heater was maintained at approximately 50 °C from 200 cycles to 2000 cycles with a slight fluctuation, and the initial increase in the temperature might have been caused by detachment of some of the unconnected Ag NWs, which led to a higher resistance of the heater. In contrast, the output temperature of the bare Ag NW-based heater constantly increased during the bending test until breakdown after 500 cycles. Considering the requirements of long-term stability under high-current and temperature stability under mechanical bending for application in high-performance power devices, heaters or solar cells, the flexible transparent electrodes made from Ni-enhanced Ag NWs networks are highly promising materials.

### Enhanced Joining Mechanism of Electroplating

**Figure 5:**
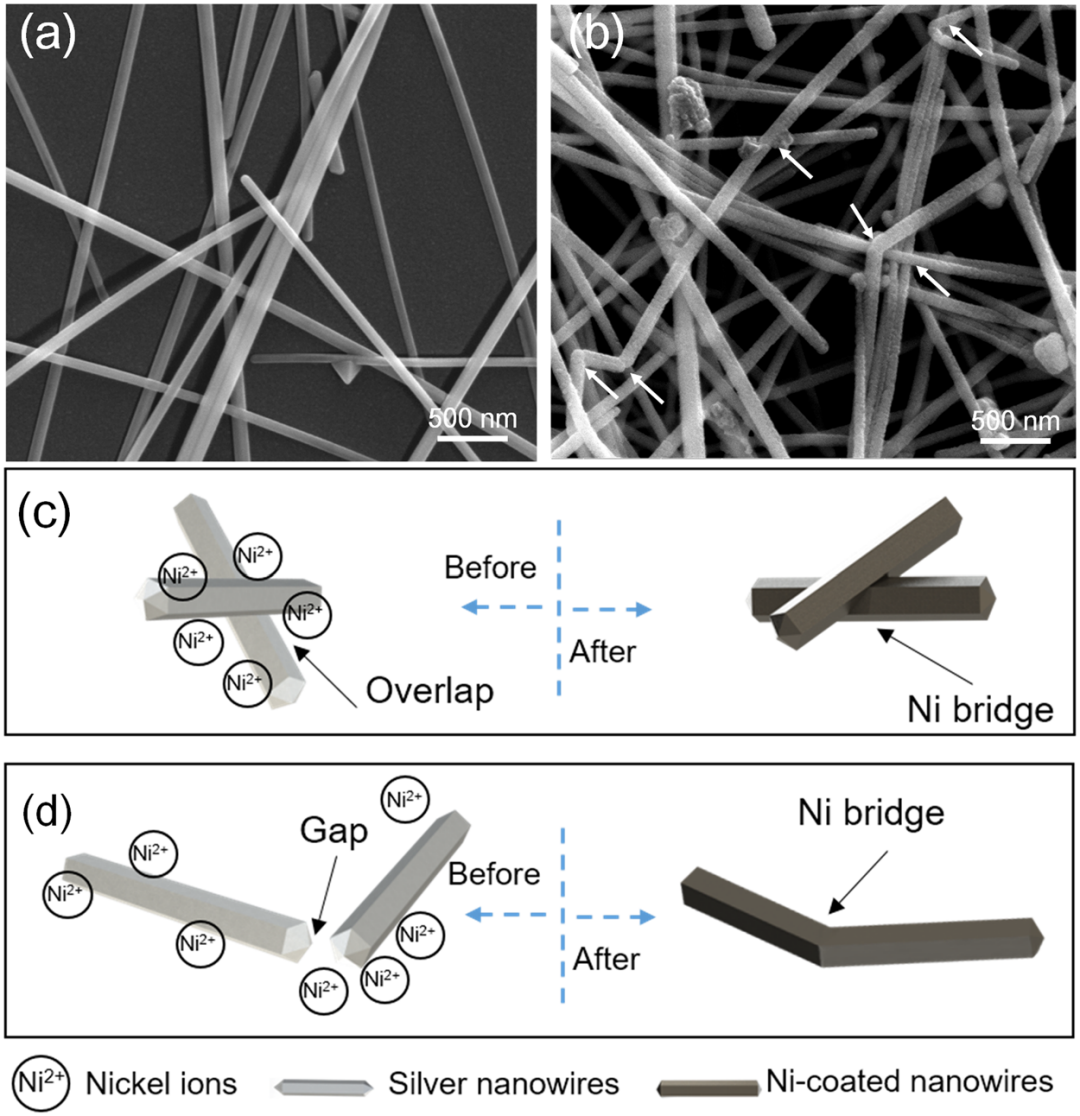
SEM images of (**a**) the bare Ag NWs and (**b**) the Ni-coated Ag NWs. Schematics of the Ni ion bridge joining mechanism for X- (**c**) and Y-shaped junctions (**d**).

The cohesive performance of the Ag NW networks was improved after completion of the modified electroplating method. For application in the typical heating method, the junction between Ag NWs should be of good quality because the Ag NW network will otherwise be nonuniform. The variable connection conditions in the nonuniform network would lead to heterogeneous conduction in the treated films, which would increase the operational difficulty. Unlike in the simple heating method, dipping the Ag NW network in an electroplating solution could achieve a lower contact resistance in an ionic solution than in air. Moreover, this method could avoid electroplating of the areas around the Ag NW network, which is unavoidable for most coating methods. In general, ultra-long Ag NWs are critical for improving the transparency and conductivity of the nanowire networks^28,29^. However, the loose connections between Ag NWs decrease the performance of the Ag NW network, as shown in Fig. 5a. After the electroplating process, the gaps between Ag NWs were filled with Ni. As the Ni shell became thicker, some adjacent Ag NWs were connected by Ni, as indicated by the arrows in Fig. 5b. At the loose connections between two Ag NWs, the Ni ions in the solution formed an ion bridge that enhanced the conductivity of the junction. When the Ag NW network was utilized as a cathode, the Ni ions were reduced to Ni atoms on the surface of the Ag NWs, the gaps between Ag NWs were filled by the reduced Ni atoms. Figure 5c,d illustrate two typical junctions formed from overlapping and head to head interactions, respectively between two Ag NWs. However, the diameter of the Ag NWs also increased because of Ni ion reduction.

The newly added Ni coating clearly increased the conductivity of the Ag NW films, but also declined the optical performance. Fig. S2 shows the UV-Vis spectra curves of the Ag NW transparent films before and after electroplating, respectively. For the bare Ag NW-based film, the surface plasmon resonance peaks of the Ag NWs appeared at 354 nm and 396 nm, corresponding to the out-of-plane quadrupole resonance and out-of-plane dipole resonance modes, respectively^30,31^. In contrast, the plasmon resonances were suppressed and broadened after Ni was coated on the surface of the Ag NWs. This observation was in good agreement with the findings for Ag-Ni nanoparticles^32^. There were two possible reasons for the observed decrease in transparency. First, the transparency of NW films is commonly understood depend on the percentage of “voids” between Ag NW network. The light passed through the transparent substrates without obstruction of Ag NWs at the void area. After electroplating, the void area decreased with the increase in the NW thickness, which led to the decrease in transparency^23^. Another reason is that the plated Ni shell increased the roughness of the NWs after electroplating, which caused more incident light to be scattered or absorbed by the electroplated NWs^33^.

### Growth Mechanism of the Ni Shell

According to electroplating theories, the amount of Ni deposited on the Ag NW cathode was proportional to the product of the current and time^34^, as follows:

1 $$m=1.095\times aIt$$

where *m* is the amount of Ni deposited on the cathode in grams, *I* is the electroplating current in amperes, *t* is the electroplating time in hours, and *a* is the current coefficient. The constant (1.095) in grams per ampere hour is calculated from Faraday’s laws. This equation shows that three major parameters (*I, t* and *a*) influence the final product. The influences of the electroplating current and time are straightforward, but that of the current coefficient is complicated and involves in many factors, including the electrode shape, electrode distance, solution conditions, and pH of the solution.

**Figure 6:**
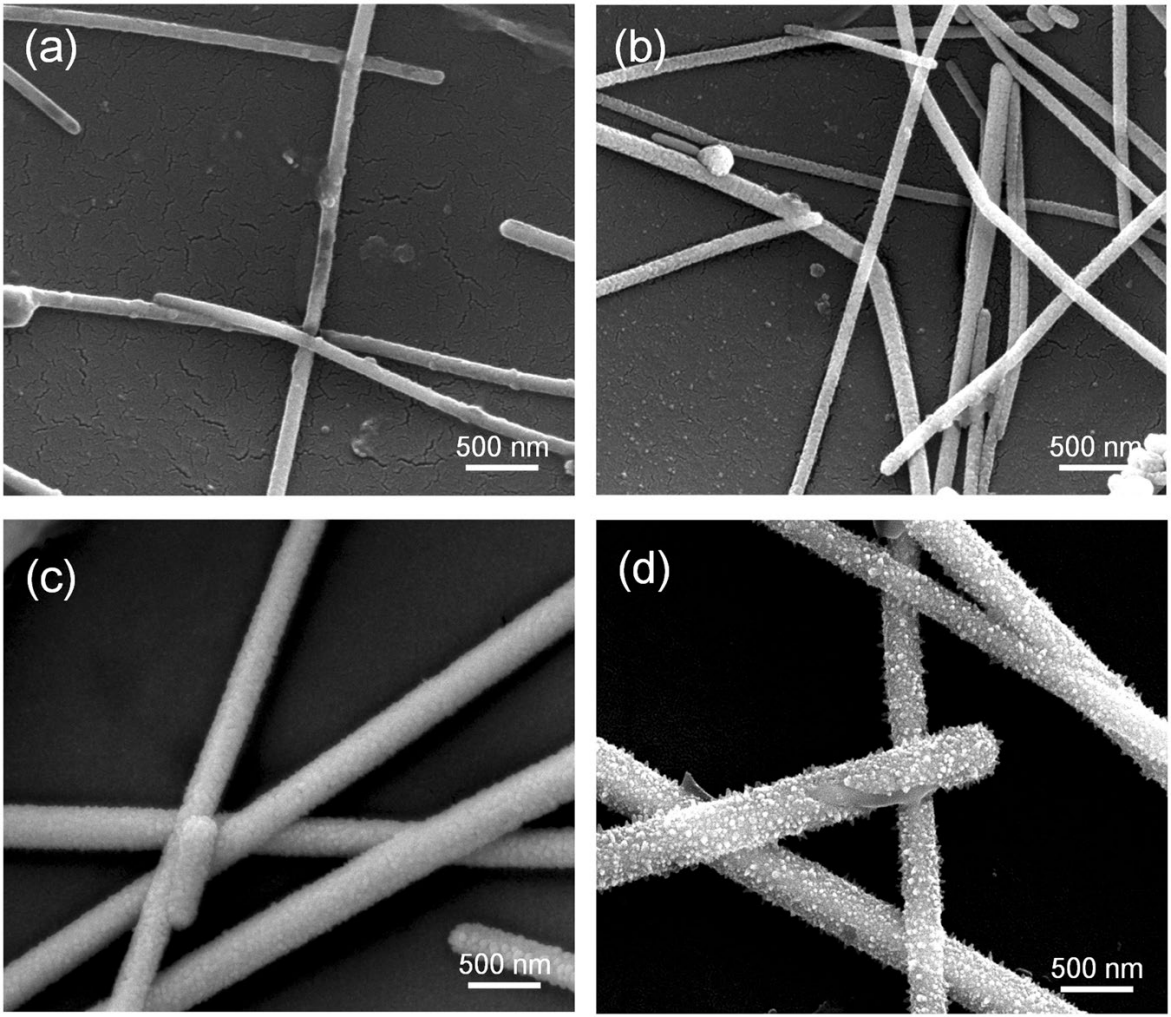
Morphologies and structures of the Ag NW networks subjected to the Ni electroplating process at 0.2 A/dm^2^ for (**a**) 2 s, (**b**) 10 s, (**c**) 20 s and (**d**) 30 s.

To investigate the growth of the Ni coating, the Ag NWs were electroplated for various times while holding the other parameters constant, as described in the experimental procedures. The thickness of the Ni coating strongly affects the transparency, electrical resistance and maximum temperature of the Ag NW film. Therefore, these three characteristics are usually used to measure the growth of the Ni coating. Table 1 lists the characteristics of the Ag NW electrodes coated at different electroplating times. Figure 6 shows the scanning electron microscopy (SEM) images of the Ag NW networks electroplated at 0.1 A for 2 to 30 s. With the increase in the electroplating time, the Ni coating became thicker, and the transparency of the Ag NW film decreased accordingly. Additionally, the deposition of Ni led to the formation of tight connections between nanowires and an increase in the cross-sectional area of the Ag NWs, thereby dramatically decreasing the electrical resistance of the sheet. Note that the maximum temperature of the electrode sheets electroplated for longer than 20 s reached 180 °C. At that temperature, the PET substrate deformed and partially melted as shown in Fig. S3. Hence, the saturation temperature of the electrode was approximately 180 °C. The saturation temperature could be even higher if the substrates with higher melting points are used.

**Table 1:** Thermal response characteristics at the maximum temperature of the samples.

Electroplating time (s)	Transparency (%)	Sheet resistance (Ω·sq^−1^)	Max temperature (°C)
0	90	<200	111
2	87	100.2	123
10	80	9.4	175
20	60	1.2	180
30	45	0.5	180

According to Equation (1), the relationship between the thickness of the Ni coating and the electroplating time should be linear, but the results showed a clearly nonlinear relationship (Fig. S5a). A possible reason for this discrepancy was that the density of the Ni shell was originally high and then decreased with the continued growth of Ni^35,36^. Thus, the mass of Ni should linearly increase with increasing electroplating time. To ensure the accuracy of the results, another sample was plated for 30 s and examined by EDS. The EDS analysis results provided in Fig. S4 confirmed the hypothesis. Because the amount of Ag NWs in each sample was the same, the Ni/Ag ratio could be used to calculate the Ni mass. The ratios were determined to be 0.375, 0.515,1.326 and 1.941 for the samples electroplated for 2 s, 10 s, 20 s and 30 s, respectively. By fitting the Ni mass and electroplating time data using Origin^TM^ software, the intercept and slope were determined to be 0.122 and 0.059, respectively. The correlation coefficient, R, was 0.944, which indicates that the two parameters had a good linear relationship (Fig. S5b) and confirmed that the Ni growth rate also followed Faraday’s laws.

### Fabrication of High Thermal Performance Transparent Heaters

**Figure 7:**
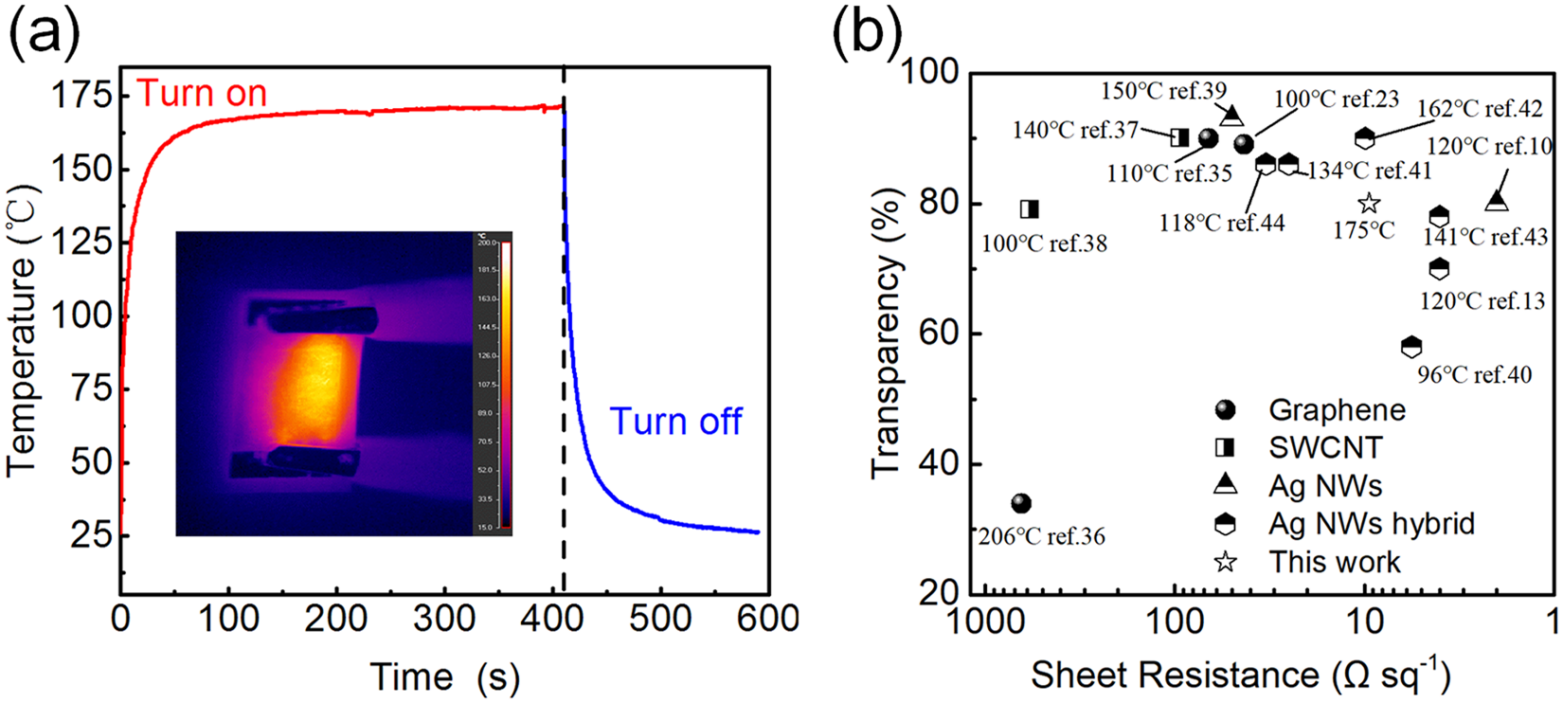
(**a**) On-off thermal response curve of the Ag NW heater. (**b**) Plot of the transparency vs. sheet resistance for the Ag NW heater with the saturation temperature indicated. The data for other nanostructured conducting materials were taken from the literature and replotted for comparison^38–47^.

Figure 7a depicts the thermal response performance of the Ni-enhanced Ag NW heater. The size of the heater was 2 cm × 1.5 cm. The temperature response of the heater was measured by an infrared camera, as shown in the inset of Fig. 7a. At a bias of 5.0 V, the temperature of the heater increased from 25 °C (room temperature) to 170 °C, within approximately 50 s. Then, the temperature was maintained at a steady state while a voltage was applied. The curve illustrated that the Ni-enhanced Ag NW-based heater possessed a good thermal response. Figure 7b compares our Ni-enhanced Ag NW TH with previously studied THs, such as those based on graphene, carbon nanotubes (CNTs), Ag NWs and Ag NW hybrids. The plot of the transmittance versus sheet resistance shows that the Ni-enhanced Ag NW TH had a relatively high performance (low sheet resistance with a high saturation temperature) comparable to that of other reported THs. Moreover, the performance of the Ni-enhanced Ag NW network could be tuned by adjusting the electroplating time to produce electrodes for different applications. The radar chart provided in Fig. S6 shows the parameters of three electrodes produced under different electroplating times (0 s, 10 s and 30 s).

## Conclusions

Highly conductive and transparent Ni-enhanced Ag NW networks with potential as high-performance heaters were obtained using a modified electroplating method. The Ni ion bridge created in the plating solution was an important medium for forming uniform and tight connections between the Ag NWs, and the newly formed Ni coating reduced the contact resistance of the Ag NW networks. High-performance Ni-enhanced Ag NW transparent film with an optimally balanced optical transparency (80% at 550 nm) and sheet resistance (9.4 Ω sq^−1^) were achieved. The Ni-enhanced Ag NW based heaters were produced to keep long-term operational stability over 120 min under high current (200 mA) and temperature stability under mechanical bending over 3500 cycles comparing with bare Ag NWs heaters. Thanks to the high thermal, electrical and mechanical stability, the Ni enhanced Ag NW transparent films were expected to apply as wearable heaters, high power devices in the near future.

## Methods

### Preparation of Ag NW Films

Ag NWs (L = 80 μm, D = 80 nm) were synthesized by a modified polyol method in our previously reported methods^37^. The as-prepared Ag NWs were dispersed in DI water at a concentration of approximately 4 mg/mL. After that, 200 μL Ag ink was filtered through a nitrocellulose membrane aided by a vacuum filtration system to fabricate Ag NW network. Then, the collected Ag NW networks were transferred onto a PET film (4 × 4 cm^2^) through a pressing transfer process. Finally, the Ag NW films were dried at 50 °C for 30 min in the oven for further tests.

### Electroplating of Ni

1 M nickel dichloride (NiCl_2_·6H_2_O), 0.5 M orthoboric acid (H_3_BO_3_) and 0.5 M ethylenediamine dihydrochloride (C_2_H_10_Cl_2_N_2_) were mixed in 500 ml DI water to form plating bath, and the pH of the solution was adjusted to 4.0 by adding aqua ammonia (NH_3_·H_2_O). Before plating, the electrolyte temperature was maintained at 60 °C using a thermostatically controlled water bath. The as-prepared Ag NWs films were submerged in the solution as the cathode, and a Ni plate was used as the anode. The electroplating process was completed at a current of 0.1 A for 5~30 s (for different samples). Then the samples were rinsed thoroughly with DI water and dried at room temperature.

### Characterization

The morphologies of the Ag NWs and Ni-coated Ag NWs were characterized using SEM (Helios Nanolab 600i, FEI NanoPorts, America) with an acceleration voltage of 20 kV. TEM (JEM 2100, Japan Electron Optics Laboratory Co.Ltd., Japan) was employed to collect images at an acceleration voltage of 200 kV. The optical and electrical properties of the Ag NW films on PET were measured using a UV/vis spectrophotometer (UV1600PC, Shanghai Jinghua Technology, China) and a four-point probe system (MCP-T370 with ASP probe, Mitsubishi Chemical Corp., Japan), respectively. A DC power source (DPS-3005D, Zhaoxin, China) was used to test the stability under high current. The temperature curve and thermal map images were obtained with an infrared camera system (A-325, FLIR Systems, Inc., America), and the voltage curve was measured by a digital multimeter (34401 A, Keysight Technologies, America). The bending tests were conducted using a homemade bending machine (Fig. S7),and the bending speed was set as 4 s per cycle (0.25 Hz).

## Electronic supplementary material

Supplementary Information
