# Supplementary material for: Cohesively enhanced electrical conductivity and thermal stability of silver nanowire networks by nickel ion bridge joining
Source: Sci Rep. 2018 Mar 27;8:5260. doi: 10.1038/s41598-018-21777-0 (PMC5869588; doi:10.1038/s41598-018-21777-0)
Supplement: Supplementary file 1 — Supplementary Information [file 41598_2018_21777_MOESM1_ESM.pdf]

Supporting Information

Cohesively enhanced electrical conductivity and thermal stability of silver nanowire networks by nickel ion bridge joining

Shang Wang, Yanhong Tian, Chunjin Hang, Chenxi Wang*

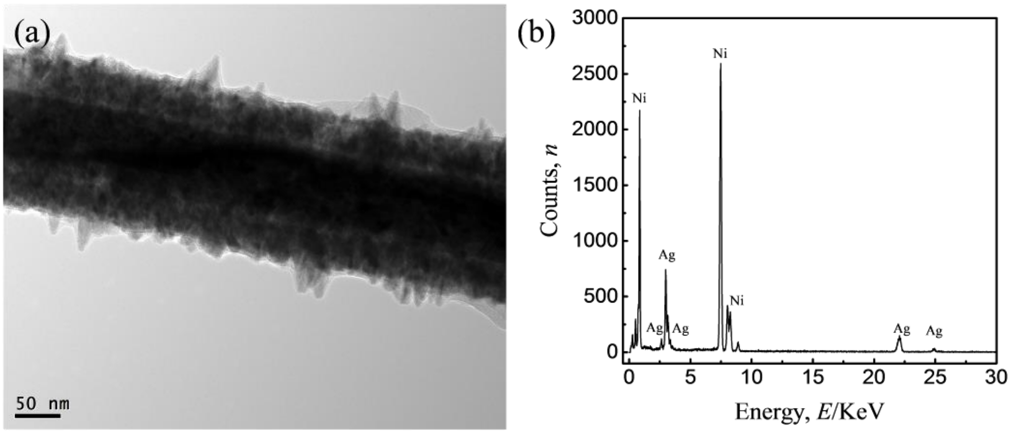

Figure S1. (a) TEM images and (b) EDS analysis results of nickel electroplated Ag NWs.

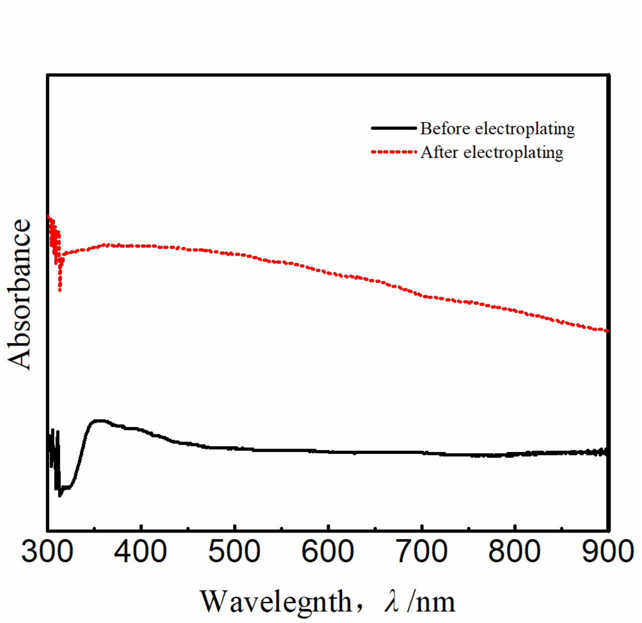

Figure S2. The UV-Vis spectra of Ag NW films before and after electroplating.

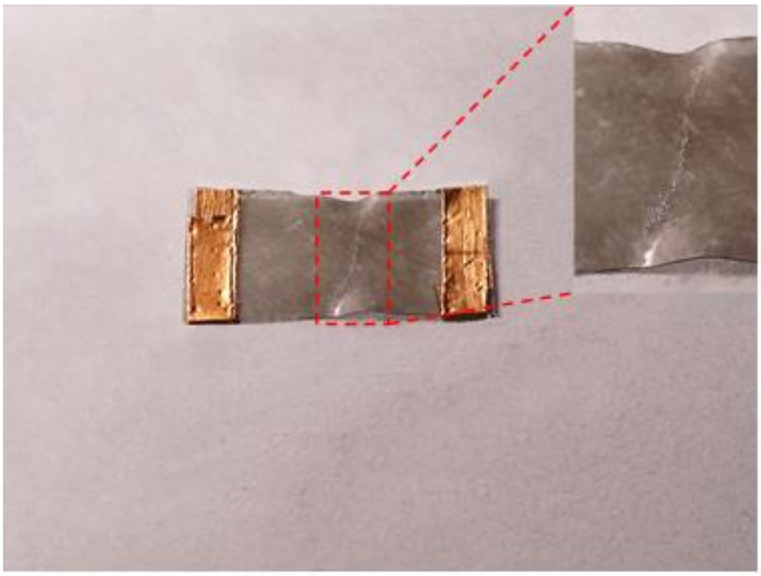

Figure S3. PET substrate melted under 180℃.

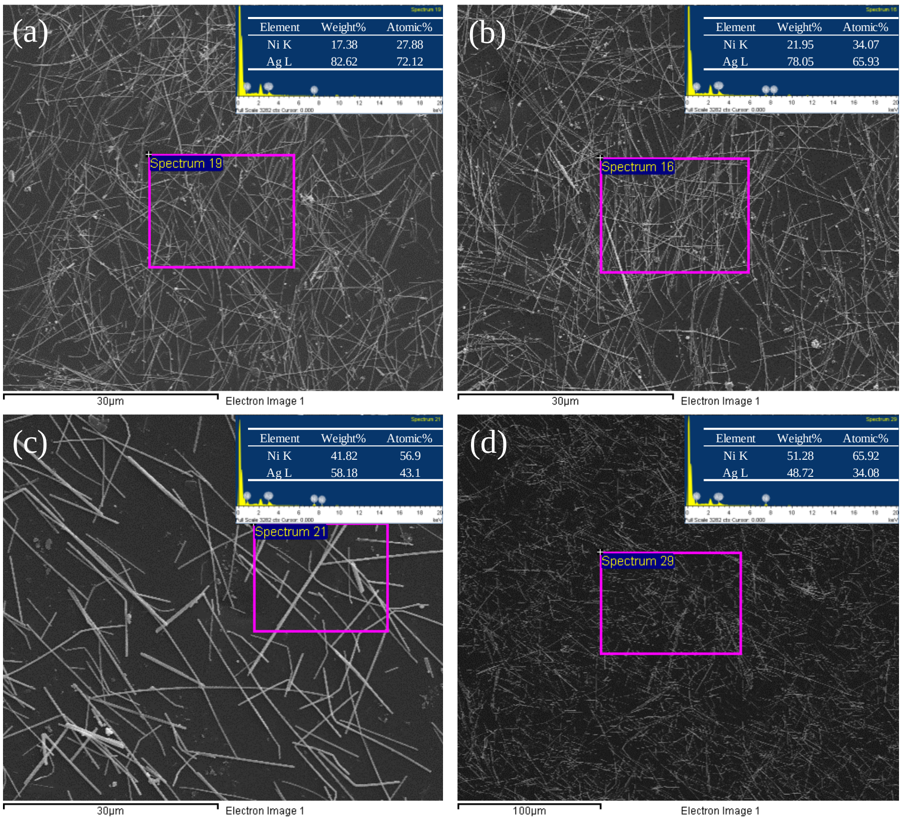

Figure S4. SEM images and EDS analysis results of nickel electroplated Ag NWs in different times. (a) 2 s with Ni/Ag ratio 0.375. (b) 10 s with Ni/Ag ratio 0.515. (a) 20 s with Ni/Ag ratio 1.326. (a) 30 s with Ni/Ag ratio 1.941.

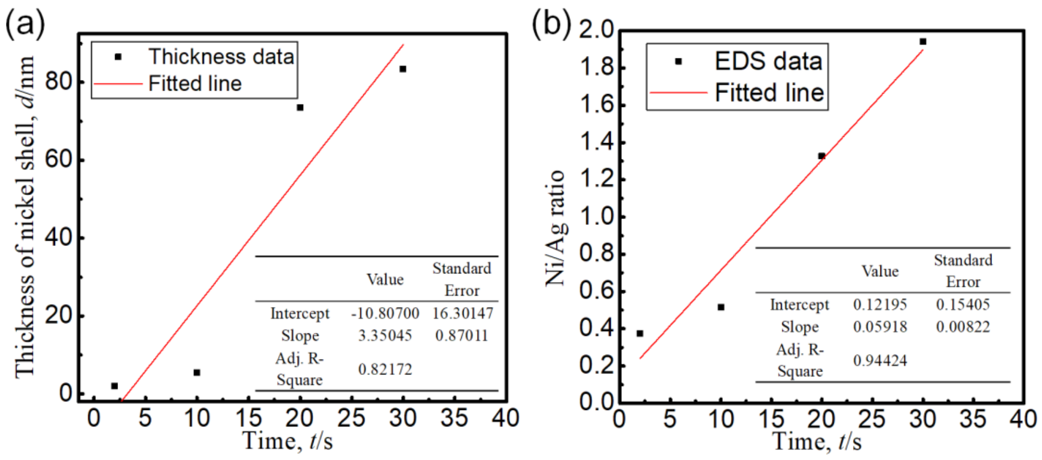

Figure S5. (a) Fitted line of electroplating time and thickness of nickel shell. (b) Fitted line of electroplating time and Ni/Ag ratio.

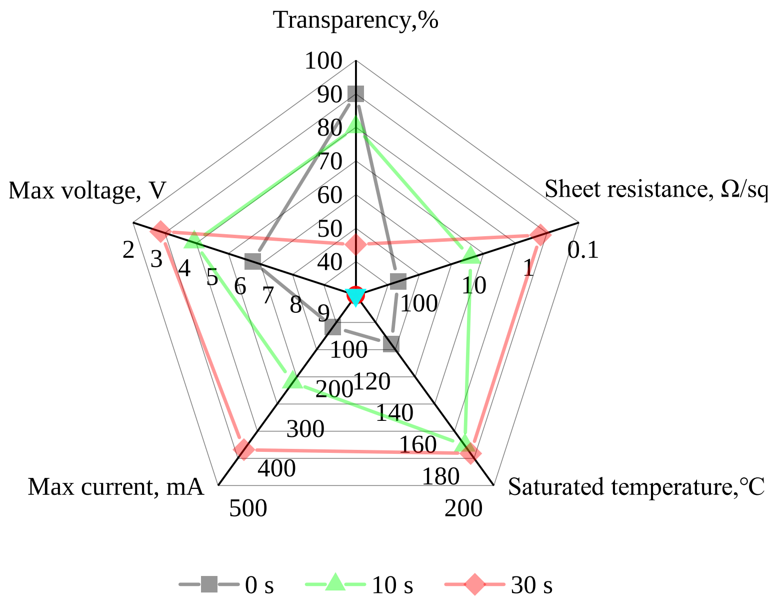

Figure S6. Key parameters of Ag NW electrodes electroplated under 0 s, 10 s, and 30 s.

For bare Ag NWs electrodes, the high sheet resistance (over 200 Ω sq^{-1}) and low saturated temperature (111℃) prevents its further applications in high temperature and large current field, although it presents good transparency. For electrodes plated for 10 s, the sheet resistance (9.4 Ω sq^{-1}) was much lower than bare electrodes, and the transparency slipped from 90% to 80%. However, the transparency of electrodes plated for 30 s was lowest than other electrodes (45%) although they reach the lowest sheet resistance of 0.5 Ω sq^{-1}. The saturated temperature and maximum temperature were also increased with the electroplating

time increasing. The changing of the performance of those electrodes illustrated that the nickel electroplating method extended the bare Ag NWs electrodes from flexible transparent electrodes to high-power and high-reliability applications, such as solar cells, transparent heaters, and power devices.

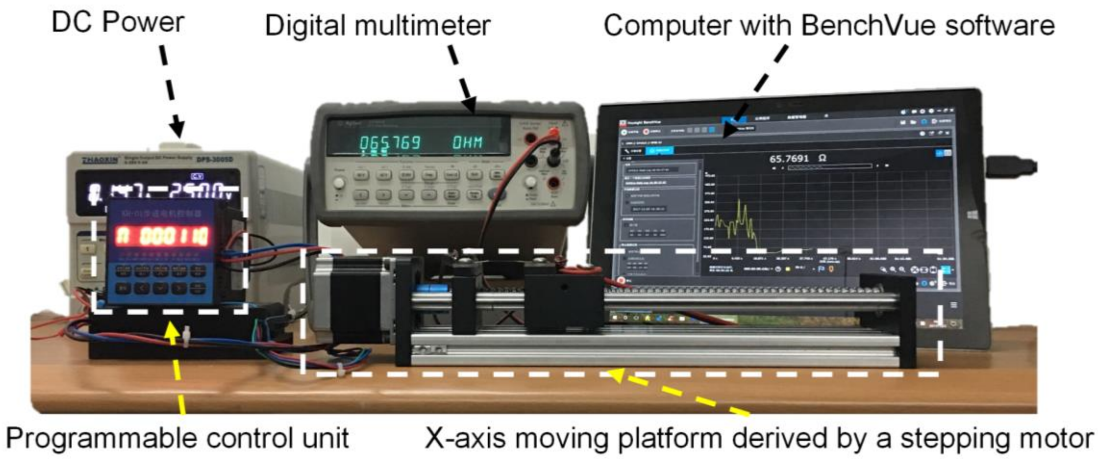

Figure S7. Bending test devices for the measuring of mechanical stability.

The home-made bending test device is shown in Fig. S7. For mechanical part, it included a DC power (DPS-3005D, Zhaoxin, China) for stepping motor (ST57H5605, Nanotech, Germany), a programmable control unit (KH-01, Yuandi Technology, China) to control the move direction and speed of the stepping motor, and a guide screw connecting (with a return difference of 0.05 mm) with the stepping motor to bend the film. For measuring part, copper tapes were pasted on the edge of Ag NW film as electrodes. The resistance of films was measured by a digital multimeter (34401A, Keysight Technologies, America) and the data was recorded by a computer equipped with BenchVue® software. Bending test parameters includes bending speed, bending radius, and bending cycle that could be controlled by the programmable control unit. The bending speed was set as 4 s per cycle (0.25 Hz). The bending radius was fixed at 2.5 mm by setting the minimum distance between clamps manually.
